# Right Ventricular Pacing and Sensing Function in High Posterior Septal and Apical Lead Placement in Cardiac Resynchronization Therapy

**DOI:** 10.1016/s0972-6292(16)30458-2

**Published:** 2012-01-31

**Authors:** HM Kristiansen, T Hovstad, G Vollan, S Faerestrand

**Affiliations:** 1 Department of Heart Disease, Haukeland University Hospital, Bergen, Norway; 2 Institute of Medicine, University of Bergen, Bergen, Norway

**Keywords:** CRT, Right ventricular pacing, Threshold, Sensing, Impedance, Complication

## Abstract

**Background:**

The conventional right ventricular (RV) lead position in cardiac resynchronization therapy pacemakers (CRT-P) is the RV apex (RV-A). Little is known about electrophysiological stability and associated complications of pacing leads in RV high posterior septal (RV-HS) position in CRT-P.

**Methods:**

Two hundred and thirty-five consecutive CRT-P patients were included from 1999-2010. Pacing thresholds at 0.5ms and 2.5V, sensing electrograms and lead impedances were measured at implant and repeated 1,3,6,12,18 and 24 months after CRT-P. Electrophysiological measurements of leads located in RV-A and RV-HS were analyzed retrospectively. Bipolar RV leads were used, including high impedance leads, passive fixation and active fixation.

**Results:**

RV pacing leads were implanted in RV-A (n=79) and RV-HS (n=156). Average RV pacing thresholds from CRT implant procedure to 24-month follow-up at 0.5ms were 0.77±0.69V in RV-A and 0.71±0.35V in RV-HS (P=0.31), and at 2.5V were 0.06±0.08ms in RV-A and 0.07±0.05ms in RV-HS (P=0.12). Average RV electrogram amplitudes from baseline to 24 months after CRT were 15.3±6.9mV in RV-A and 12.1±6.0mV in RV-HS (P=0.55). Average RV impedances during follow-up were 850±286Ω in RV-A and 618±147Ω in RV-HS (P=0.57). Similar RV lead revisions between RV-A and RV-HS were observed after 2-year follow-up (P=0.55).

**Conclusion:**

The RV-HS lead position demonstrated stable and acceptable long-term pacing and sensing function, with rates of complications comparable to conventional RV-A lead position in CRT. The RV-HS lead position is feasible in CRT-P.

## Introduction

Cardiac resynchronization therapy (CRT) is the recommended treatment in symptomatic heart failure (HF) patients on optimal medical treatment with left ventricular (LV) systolic dysfunction and prolonged QRS duration.[1-3] The conventional right ventricular (RV) lead placement in CRT has been at the RV apex (RV-A).[4] Recent studies on the detrimental effects of RV-A pacing on LV function in treating bradyarrhythmias has increased the interest in alternate RV pacing sites.[5,6] The electrophysiological performance and associated complications of selective site pacing in the interventricular septum of the RV outflow tract (RVOT) and RV mid septum has been studied in patients with a bradycardia indication for permanent pacing.[7,8] In CRT, several studies have demonstrated the long-term electrophysiological stability and associated complications of conventional lead placement.[9-12] Alternate RV lead position in CRT has demonstrated similar hemodynamic effects as RV-A in previous studies.[13-16] However, there are few data concerning long-term electrophysiological stability and associated complications in alternate RV pacing sites in CRT.

We hypothesized that a RV high posterior septal (RV-HS) lead position in CRT would provide similar long-term pacing and sensing stability as compared to the conventional RV-A. Thus, the aim of this study was to compare the electrophysiological performance of RV-HS lead position in CRT pacemakers (CRT-P) to previously implanted RV-A in a two-year follow-up. The challenges encountered with the RV-A and RV-HS lead implant procedure and need for lead revisions during the first two years were investigated.

## Methods

### Patient Population

In this study, 237 consecutive HF patients referred to CRT pacemaker (CRT-P) implantation were included between 1999 and 2010. Inclusion criteria were HF patients on optimal medical treatment in New York Heart Association (NYHA) functional class III-IV, LV ejection fraction (LVEF) ≤ 35%, LV end-diastolic diameter ≥ 5.5 cm and QRS duration ≥ 120ms. The regional ethics committee approved this study, and the patients were included after informed consent.

### Study Protocol

RV pacing thresholds at 0.5ms and 2.5V, respectively, sensing R-wave amplitudes and lead impedances at 5.0V pacing were measured during the pacemaker implantation. All measurements were repeated at follow-up 1, 3, 6, 12, 18 and 24 months after CRT. Pacemaker testing was performed by using Medtronic CareLink® pacemaker programmer (Medtronic, Minneapolis, MN, USA). Patients in need of lead revisions re-started the same routine follow-up intervals.

### CRT Implantation Procedure

**Figure 1:**
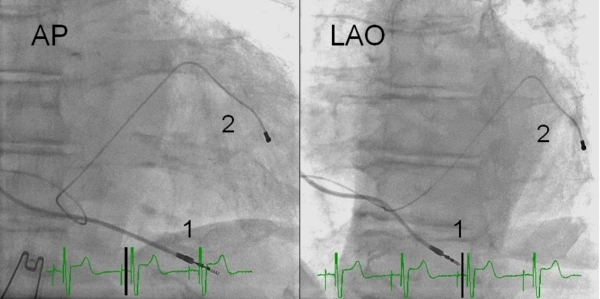
RV-A lead (1; Medtronic 4076) placement in fluoroscopic anteroposterior (AP) and left anterior oblique 30º view (LAO). The coronary venous lead (2; Medtronic 4193) is also shown. RV-A = right ventricular apical lead placement.

**Figure 2:**
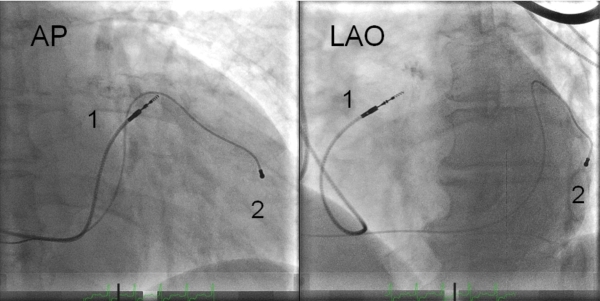
RV-HS lead (1; Medtronic 4076) placement in flouroscopic anteroposterior (AP) and left anterior oblique 30º view (LAO). The coronary venous lead (2; Medtronic 4193) is also shown. RV-HS = right ventricular high posterior septal lead placement.

A CRT pacing system was implanted under local anaesthesia with a transvenous introduction of the leads via the left cephalic or subclavian vein using different bipolar RV leads (Table 1). Two experienced implanters (SF,TH) performed the CRT-P implant procedures. Both CRT implanters placed the RV leads in RV-A and RV-HS equally distributed. During the first six years of CRT implantation the conventional RV-A lead placement was used at our center. The RV-HS lead placement was selected in the majority of the patients from 2005 although the choice of lead position was left to the discretion of the implanting physician. The RV lead positions were achieved guided by fluoroscopy using three standard views as a routine: anteriorposterior view (AP), left anterior oblique 30º view (LAO) and right anterior oblique 30º view (RAO). The RV-A lead position was defined as the distal part of RV lead close to or below the silhouette of left diaphragm visualized in the AP fluoroscopic view.[17] In LAO view RV-A position was demonstrated by a straight or slightly curved anterior lead direction (Figure 1). In RAO fluoroscopic view the RV-A position was confirmed by an anterior direction of the leads with the tip electrode close to the inferior part of the sternum. This view was also used primarily to avoid lead positioning in RV free wall or in the middle or lateral cardiac vein. The RV-A lead position was achieved by using conventional bipolar RV pacing leads (n=79) with a stylet. RV-HS lead placement was defined positioning in the inferior part of the RVOT in AP fluoroscopy view with a posterior lead tip orientation in fluoroscopic LAO view [17] (Figure 2). The RAO fluoroscopic view was used to ensure that the RV lead was not positioned in the coronary sinus (CS) or great cardiac vein. For the first consecutive 107 patients, the RV-HS lead position was obtained by using the Select Secure systemTM (Medtronic, Minneapolis, MN, USA) with deflectable guide catheter. For the next 49 patients, conventional bipolar RV pacing leads with preshaped stylets were used.

The first method to achieve the RV-HS lead position was a catheter based lead delivery system.[17] The deflectable guide catheter was tracked over a guidewire into the RV before the guidewire was removed and replaced by the lumenless RV lead. The lead was advanced to near the catheter tip and the guide catheter was then deflected and positioned to the inferior part of RVOT and to the posterior part of interventricular septum by counterclockwise rotation of the catheter. After reaching the desired implant site, the lead was advanced to the myocardium and fixated by the tip screw, and the deflectable catheter carefully removed. The RVOT position was verified by AP view, and posterior pointing verified in LAO view.

The second method of obtaining the RV-HS lead position was by using a conventional pacing lead manoeuvred by preshaped stylets.[19] First, the lead was advanced to the pulmonary artery facilitated by a curved stylet. Next, a second preshaped stylet resembling a swan neck was introduced to the lead tip. This stylet had a generous primary curve and a terminal 2cm bend to form a secondary curve. The secondary swan neck curve was further modified to make the terminal 2cm of the stylet point in a posterior direction. The RV lead was carefully pulled back from the pulmonary artery during slight counterclockwise rotation of the stylet to facilitate a posterior direction of the lead tip to move to the posterior interventricular septum of the RVOT. The position of the lead tip pointing posteriorly was verified in the LAO view before the lead was fixed by the tip screw to the myocardial wall.

The CS leads were placed in a lateral or posterolateral CS vein tributary from AP and LAO view.20 The right atrial leads were positioned in right appendage demonstrated by an anterior and upward position of the distal part of the in the RAO view.[17]

### Statistical Analysis

Statistical analyses were performed using SPSS software version 18.0 (SPSS, Chicago, IL). All variables are expressed as mean ± SD. Continuous variables were compared by using Student t-test. Time-related repeated measurements and were compared by ANOVA for repeated measurements with Greenhouse-Geisser correction. Nonparametric data were analyzed by Pearson χ^2^ or Fisher's exact test. P-value of < 0.05 was considered statistically significant.

## Results

### Patient population

Out of 237 patients referred for CRT-P, 158 were selected for RV lead placement in RV-HS position. In 2 patients we failed to achieve this lead position (1.3% failure rate), and both were successfully repositioned to RV-A, and were excluded from further analysis. Accordingly, the study group consisted of 235 CRT-P patients: 79 patients with RV-A lead position and 156 with RV-HS lead position. Baseline characteristics of the study patients are listed in Table 2.

### Pacing Thresholds

**Figure 3:**
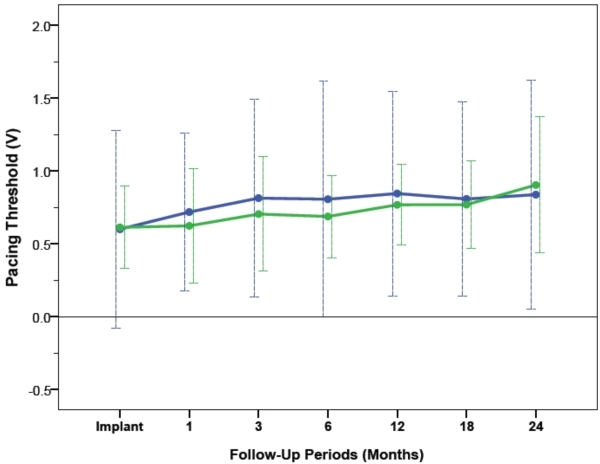
Pacing thresholds at 0.5ms pulse duration measured at baseline and at different follow-up periods between RV-A (blue) and RV-HS (green). Abbreviations as in Figure 1 and 2

**Figure 4:**
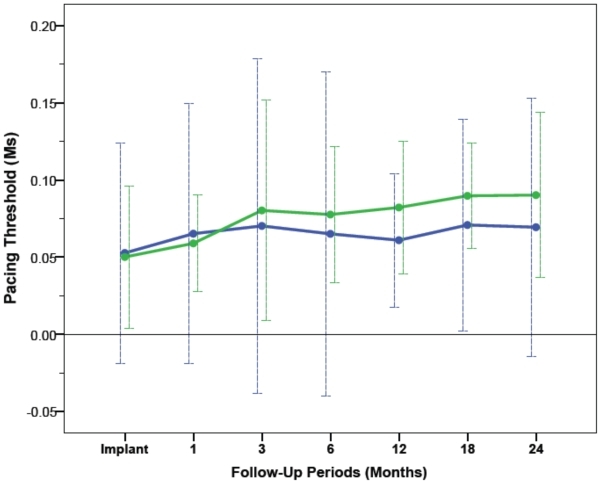
Pacing thresholds at 2.5V measured at baseline and at different follow-up periods between RV-A (blue) and RV-HS (green). Abbreviations as in Figure 1 and 2

The average voltage pacing thresholds and pulse duration pacing threshold at 0.5ms and 2.5V respectively, in RV-A and RV-HS, are demonstrated in Figure 3 and 4. From implant to 24 months after CRT, the RV average voltage pacing threshold at 0.5ms remained stable at 0.77±0.69V in RV-A (P=0.09) and 0.71±0.35V in RV-HS (P=0.31). From baseline to 24-month follow-up, the average RV pulse duration pacing threshold at 2.5V was stable at 0.06±0.08ms in RV-A (P=0.43) and 0.07±0.05ms in RV-HS (P=0.12). The average pacing threshold from CRT implantation to 24 months after CRT were similar between RV-A and RV-HS, measured at 0.5ms (p=0.43) and at 2.5V (p=0.12).

### Sensing Electrogram Amplitudes and Lead Impedances

**Figure 5:**
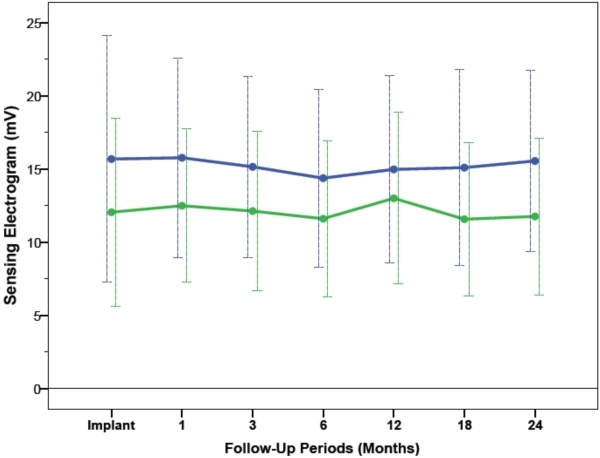
Sensing amplitude threshold measured at baseline and at different follow-up periods between RV-A (blue) and RV-HS (green). Abbreviations as in Figure 1 and 2

**Figure 6:**
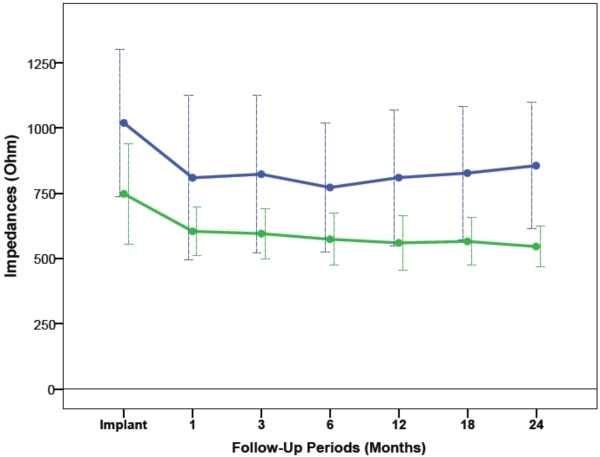
Lead impedances measured at baseline and at different follow-up periods between RV-A (blue) and RV-HS (green). Abbreviations as in Figure 1 and 2

The average sensing electrogram amplitudes and lead impedances from RV-A and RV-HS are illustrated in Figure 5 and Figure 6. From implant to 24 months after CRT, the average sensing amplitudes were stable at 15.3±6.9mV in RV-A (P=0.55) and 12.1±6.0mV in RV-HS (P=0.78), and were comparable between the two RV lead positions (P=0.55). From baseline to 24-month follow-up, the lead impedances decreased and averaged at 850±286Ω in RV-A (P=0.003) and 618±147Ω in RV-HS (P<0.001), but were not different between the RV-A and RV-HS lead position (P=0.57).

### Operative observations

Operative observations are listed in Table 3. The main operative challenges for both RV lead positions were either high pacing threshold or low R-wave amplitudes that required repositioning of the RV lead. Repositioning procedures of the RV lead was targeted towards finding a location as close as possible to the original implant site with acceptable pacing and sensing function. In 8 patients (RV-HS; n=6) it was difficult to achieve the anatomically appropriate RV lead positions and several lead position attempts were needed to obtain the targeted site. There were 4 intraoperative lead dislodgements (RV-HS; n=3) that were successfully corrected during the primary operation. No major complications related to RV lead placements were observed.

### Late Lead Revisions

Out of the 120 patients with RV-HS lead placement that reached two-year follow-up (lead model 3038; n=107; lead model 4076; n=13), RV lead revisions were necessary for 3 patients. They all had the lead model 3830. For one patient, lead revision was done after 3 days due to loss of pacing capture without lead dislodgement. For the other two patients, revisions were completed after 106 days and 491 days, respectively, due to lead dislodgements. Of the 60 patients with RV-A lead positions that reached two-year follow-up, no RV leads had to be revised. The lead revisions performed in the two RV lead positions two years after CRT were similar (P=0.55).

## Discussion

The main finding of this 2-year follow-up comparing the electrophysiological performance of alternate RV lead position in CRT-P can be summarized as follows: 1) similar and stable pacing and sensing function in both RV-A and RV-HS during long-term CRT-P; 2) decreasing RV lead impedances during follow-up, but comparable findings between the two RV lead positions; 3) similar operative challenges and late RV lead revisions in RV-A and RV-HS.

To our knowledge, the current study is the first to compare the electrophysiological performance of alternate RV lead position in CRT-P. Previous studies have demonstrated the pacing lead stability and sensing function in RV-A as compared to alternate RV lead position in bradycardia indication for permanent pacing. Burri et al [8] studied 362 pacemaker recipients, and found similar pacing- and sensing lead performance and need for RV lead repositioning between RV-A and RVOT. The long-term stability of RV-mid septal position was reported by Medi et al [7], in 100 pacemaker patients. The current study demonstrated similar long-term electrophysiological performance between RV-A and RV-HS in CRT-P, in accordance with previous findings of alternate RV lead position in permanent pacing for bradycardia. However, the CRT patients with moderate-to-severe heart failure and remodelled hearts might be structurally different from the typical population of bradypacing. Moreover, the present study demonstrates that RV-HS lead position is feasible also in CRT-P patients, including two different methods for placement of the RV-HS lead.

RV lead impedances can decrease on long-term follow-up, but might also be dependant on RV lead characteristics. [7,8] A decrease in RV-lead impedance was observed in the current study, similar as the aforementioned studies. Different RV leads were used in the present study, including 47 of the 79 high-impedance RV-A leads [21], and this might have influenced on the electrophysiological results. The reported complications to alternate RV lead placement in bradycardia-pacing have been similar as in RV-A. [8] The present study found comparable intraoperative challenges and reinterventions between the two RV lead positions. We experienced 3 lead revisions in RV-HS and none in RV-A during two-year follow up, but not statistical significant different.

### Clinical implications

The current study demonstrates that the RV-HS lead position is feasible in CRT-P. The RV-A position in CRT is preferred in CRT, and has demonstrated long-term stability. [4] Moreover, it is essential that any alternate positions to the conventional RV-A in CRT maintain similar long-term stability. Furthermore, the influence of RV lead position on LV reverse remodelling is uncertain, but alternate RV lead position in CRT is feasible as reported in the present study. Large prospective randomized trials are needed to identify the optimal RV lead position in CRT.

### Study limitations

The present retrospective single-centre study may be limited as the there were no randomization of the RV lead positions. Furthermore, the patients were slightly different at baseline as the patients with RV-A lead position suffered from more severe HF. The study population might bee too small or the duration of the follow-up period too short to identify differences in electrophysiological lead stability or complication rates between the two RV lead positions. Different RV leads, including active and passive fixation leads, were used, with differences in pacing surface area and anode-cathode distance, which can influence the results in this study.

## Conclusion

The present study demonstrate that RV-HS lead position is feasible in CRT-P, with similar long-term pacing and sensing functions and associated complications as the conventional RV-A lead placement.

## Figures and Tables

**Table 1:**
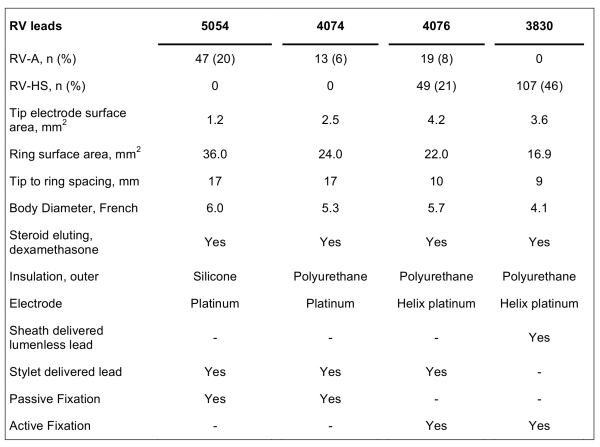
Right Ventricular Bipolar Pacing leads.*

* Medtronic, Minneapolis, MN, USA; RV-A = right ventricular (RV) apical lead position; RV-HS = RV high posterior septal lead position

**Table 2:**
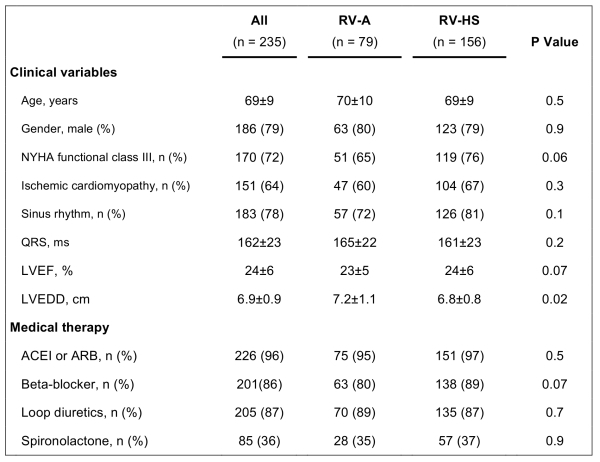
Baseline Characteristics of the Patients According to Right Ventricular Position

RV-A = right ventricular (RV) apical lead position; RV-HS = RV high posterior septal lead position; NYHA = New York Heart Association; LVEF = LV ejection fraction; LVEDD = LV end-diastolic diameter; ACEI = angiotensin-converting enzyme inhibitors; ARB = angiotensin receptor blockers

**Table 3:**
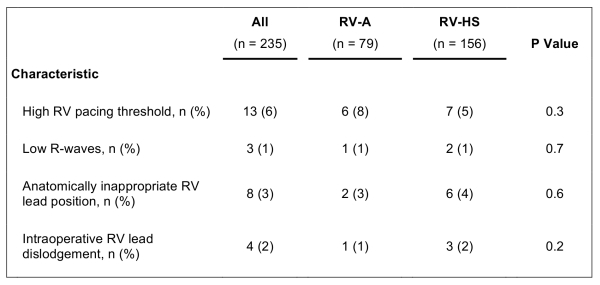
Operative Observations

RV-A = right ventricular (RV) apical lead position; RV-HS = RV high posterior septal lead position
